# A Rare Case of Hydralazine-Induced Diffuse Alveolar Hemorrhage

**DOI:** 10.7759/cureus.47591

**Published:** 2023-10-24

**Authors:** Angela Xue, Adele Bernard, Vanessa Moreno, Lindsey Phillips, Evan Raff

**Affiliations:** 1 Medicine, University of North Carolina School of Medicine, Chapel Hill, USA; 2 Pathology and Laboratory Medicine, University of North Carolina School of Medicine, Chapel Hill, USA

**Keywords:** anti-neutrophil cytoplasmic antibody (anca)-associated vasculitis (aav), pulmonary renal syndrome, pauci-immune crescentic glomerulonephritis, diffuse alveolar hemorrhage, hydralazine

## Abstract

Hydralazine-induced anti-neutrophil cytoplasmic antibody (ANCA) vasculitis may occur any time after hydralazine initiation. General internists should recognize diffuse alveolar hemorrhage (DAH) as a rare complication of this condition, as early treatment reduces the associated high risk of mortality. We describe the case of an 82-year-old female with diastolic heart failure who presented with a one-month history of worsening dyspnea on exertion and a one-week history of scant hemoptysis and fatigue. Her medications included aspirin and hydralazine. She was hypoxic with bilateral expiratory wheezes on exam. Labs showed new anemia, elevated creatinine, proteinuria, and hematuria. Chest computed tomography showed asymmetric bilateral upper lobe ground-glass attenuation superimposed on interlobular septal thickening and intralobular lines. Further testing showed anti-nuclear antibody, positive ANCA, perinuclear ANCA (p-ANCA), and anti-myeloperoxidase ANCA (anti-MPO-ANCA). Renal biopsy revealed MPO-ANCA, pauci-immune, necrotizing, and crescentic glomerulonephritis. She was diagnosed with DAH secondary to hydralazine-induced ANCA-associated vasculitis (AAV). Hydralazine is an anti-hypertensive medication with known potential for autoimmune reactions. Of these, AAV is a rare sequela mediated by anti-MPO and most commonly affects the kidneys. In rare circumstances, patients with AAV can develop pulmonary-renal syndrome, resulting in both glomerulonephritis and DAH with an associated high risk of mortality. Diagnosis requires a high index of suspicion in patients with acute kidney injury of unclear etiology. Early diagnosis through immune work-up and kidney biopsy should be pursued, as prompt recognition of the vasculitis, cessation of hydralazine, immunosuppression, and early plasma exchange are essential to an improved prognosis.

## Introduction

Hydralazine is an arterial vasodilator commonly used to treat hypertensive emergency and essential hypertension refractory to first-line anti-hypertensives, such as calcium channel blockers, angiotensin-converting enzyme inhibitors, etc. [1]. Specifically, intravenous hydralazine is frequently utilized in hospitals for non-urgent cases of hypertension, although it has been found to cause variable pressure-lowering effects and an increased risk of hypotension [2]. Oral hydralazine in combination with isosorbide nitrate has been shown to provide mortality benefits among African American individuals with heart failure with reduced ejection fraction [3].

Although generally well-tolerated, hydralazine can induce anti-neutrophil cytoplasmic antibody (ANCA)-associated glomerulonephritis (AAGN) with a rapidly progressive clinical course mixed with extrarenal symptoms such as arthralgia, pleuropulmonary disease, and cutaneous vasculitis [4,5]. Pulmonary hemorrhage is an extrarenal complication associated with an increased mortality risk; this condition is termed pulmonary-renal syndrome (PRS) [6-8]. In rare cases, hydralazine-induced AAGN can lead to diffuse alveolar hemorrhage (DAH) requiring immediate medical intervention [7,9].

In this report, we present the case of an 82-year-old woman with a one-month history of worsening dyspnea on exertion and a one-week history of scant hemoptysis and fatigue who was found to have hydralazine-induced ANCA-associated vasculitis (AAV) leading to DAH. Despite treatment with plasma exchange and cyclophosphamide, she unfortunately passed from hypoxic respiratory failure.

Hydralazine-induced AAV may occur at any time after hydralazine initiation [4]. General internists should recognize DAH as a rare but serious complication of this condition, as early treatment reduces the associated high risk of mortality.

This article was previously presented as a poster at the Society of Hospital Medicine Converge 2023 on March 28, 2023, and as an oral presentation at the American College of Physicians Internal Medicine Meeting 2023 on April 29, 2023. The corresponding abstract was published in the Journal of Hospital Medicine on June 7, 2023.

## Case presentation

An 82-year-old female presented to the emergency department with a one-month history of worsening dyspnea on exertion and a one-week history of scant hemoptysis and fatigue. Her past medical history included heart failure with preserved ejection fraction, chronic obstructive pulmonary disease, gastroesophageal reflux disease, depression, hypertension, and carotid stenosis status-post endarterectomy. She denied tuberculosis risk factors, night sweats, weight loss, fevers, or skin, joint, or urinary changes. She was fully vaccinated against coronavirus disease 2019 (COVID-19). Her medications included aspirin, hydralazine, amlodipine, amoxicillin, azelastine, carvedilol, furosemide, losartan, rosuvastatin, sertraline, and spironolactone. Her medical history and social history were otherwise unremarkable.

Upon admission, she was afebrile, hypertensive with a blood pressure of 180/68 mmHg, and hypoxic with an oxygen saturation of 85% on room air. Physical examination was significant for diffuse bilateral expiratory wheezes. Labs showed anemia (hemoglobin 9.8 g/dL with a historical baseline of 12 g/dL), an elevated creatinine (1.3 mg/dL with a historical baseline of 0.9 mg/dL), proteinuria (30 mg/dL), and hematuria (71 red blood cells per high-power field). Urine sediment showed many tubular epithelial cells along with 1-2 dysmorphic epithelial cells per high-power field without evidence of casts or acanthocytes.

Transthoracic echocardiogram showed mildly increased left ventricular wall thickness and normal function. Chest computed tomography (CT) angiography showed no pulmonary embolism but did demonstrate bilateral paracentral predominant mixed consolidative and ground-glass opacities within the posterior segment of the upper and lower lobes, favoring aspiration versus underlying infectious or inflammatory etiologies. Based on her clinical swallow evaluation, oropharyngeal dysphagia and aspiration were considered less likely.

During her hospitalization, despite aggressive diuresis, discontinuation of hydralazine, and broad-spectrum antibiotic coverage for possible community-acquired pneumonia, her oxygen requirement continued to increase, progressing to a maximum of 70% fraction of inspired oxygen via high-flow nasal cannula. Worsened hypoxia in combination with persistent hemoptysis led to the clinical suspicion of DAH secondary to an underlying inflammatory condition.

**Figure 1:**
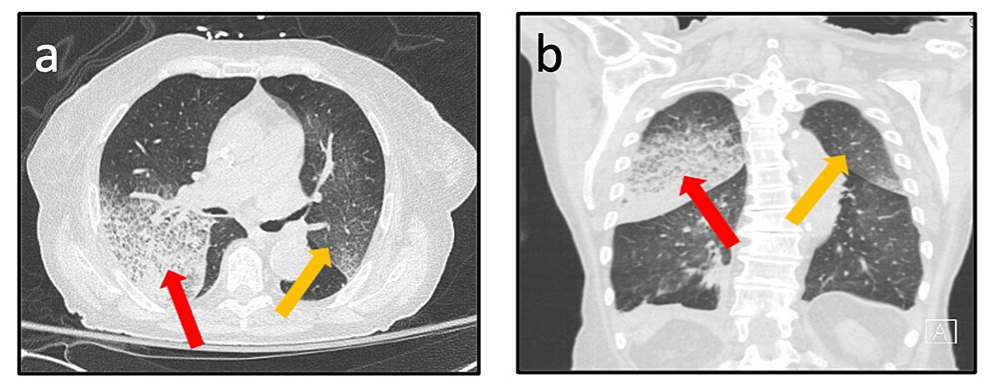
Coronal (a) and axial (b) chest CT demonstrating ground-glass attenuation superimposed on interlobular and intralobular lines in the right (red arrow) and left (orange arrow) upper lobes, an imaging pattern associated with parenchymal hemorrhage. CT: computed tomography

**Figure 2:**
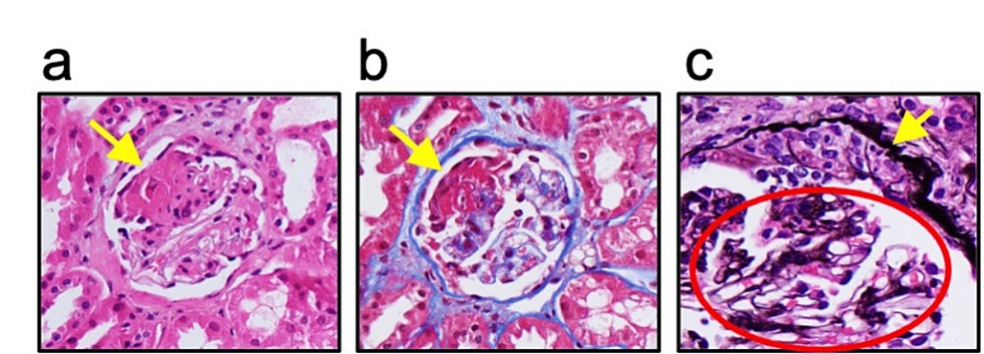
Renal biopsy demonstrating glomeruli with focal and segmental necrotizing lesions and disruption of basement membranes, best seen in (a) and (b). The necrotizing lesion is bright eosinophilic with H&E stain (yellow arrow in (a)) and bright red with trichrome stain (yellow arrow in (b)) confirming that this is fibrinoid necrosis. (c) shows glomeruli with cellular crescents characterized by extra-capillary hypercellularity seen on Jones silver stain (yellow arrow). The spared glomerular tufts have no mesangial and/or endocapillary hypercellularity (red circle). H&E: hematoxylin and eosin

Further laboratory analysis revealed positive ANCA, anti-nuclear antibody (ANA; titers of 1:640), and anti-myeloperoxidase ANCA (anti-MPO-ANCA; 65.5 U/mL, reference range <21 U/mL). Antibodies to double-stranded DNA, Smith, RNP, Ro/SSA, La/SSB, Jo1, Scl70, glomerular basement membrane, and anti-proteinase 3-ANCA (anti-PR3-ANCA) were all negative. An extensive microbiologic work-up yielded no evidence suggestive of an underlying infection. Complement 3 and 4 levels were normal. The patient's wishes were to remain "do not resuscitate/do not intubate"; thus, diagnostic bronchoscopy was deferred. She lacked the functional reserve to tolerate pulmonary function tests (PFTs). A repeat chest CT showed ground-glass attenuation superimposed on interlobular and intralobular lines in both right and left upper lobes (Figure 1), indicative of parenchymal hemorrhage. Renal biopsy revealed focal activity with cellular crescents or necrosis in at least five of 26 glomeruli, moderate fibrous thickening of the arterial intima, and mild arteriole intimal hyalinosis on light microscopy (Figure 2). Immunofluorescence staining showed focal and segmental glomerular tuft staining for fibrinogen and a pauci-immune pattern. Combined, these findings were consistent with AAGN, likely secondary to hydralazine.

In the setting of symptom onset coinciding with the start of hydralazine, positive ANCA and anti-MPO-ANCA, DAH, and necrotizing crescentic glomerulonephritis, the diagnosis of DAH secondary to hydralazine-induced AAV was established. She experienced brief but transitory respiratory improvement after the initiation of high-dose methylprednisolone. Despite the addition of plasma exchange and cyclophosphamide to her treatment regimen, her condition deteriorated, and she expired on comfort care.

## Discussion

Hydralazine-induced AAV is an autoimmune condition that can present with interstitial lung disease, pauci-immune glomerulonephritis, and cutaneous vasculitis [4,5,10]. This condition is predominantly associated with positive p-ANCA/anti-MPO-ANCA, though dual positivity of p-ANCA/anti-MPO and c-ANCA/anti-PR3-ANCA is present in 40% of cases [4]. Interestingly, although primary AAGN is pauci-immune on renal biopsy, hydralazine-induced AAV kidney biopsies often show immune deposits and mild positive staining for anti-sera specific for immunoglobulins (Ig; including IgG, IgA, or IgM) and/or complements (C1q or C3) [4]. Additionally, positive ANA, anti-histone antibodies, anti-cardiolipin IgG or IgM antibodies, and hypocomplementemia can also be present in patients with hydralazine-induced AAV [4,10].

The exact pathophysiology of hydralazine-induced AAV is unknown. However, studies have shown that patients with AAV abnormally express two autoantigens: PR3 and MPO. In patients with AAV, the CpG islands in both the PR3 and MPO genes are unmethylated, leading to disrupted gene silencing and autoantigen overexpression [11]. Separately, hydralazine has been found to be a synthetic DNA methylation inhibitor [5,12]. Thus, it is hypothesized that hydralazine may inhibit DNA methylation of PR3 and MPO genes, causing overexpression of the two autoantigens and leading to the development of hydralazine-induced AAV [5].

Symptoms associated with hydralazine-induced AAV include shortness of breath, arthralgia, myalgia, fatigue, hematuria, proteinuria, and rapidly progressive renal failure [8,10,13]. In very rare circumstances, patients with AAV can develop PRS, resulting in both glomerulonephritis and DAH with an associated high risk of mortality [6,14]. Research by Hogan and colleagues found that the relative risk of death for patients who developed PRS was 8.65 times greater than those who did not have PRS (95% CI: 3.36-22.2) [6].

Diagnosing hydralazine-induced AAV demands a heightened suspicion in cases of unexplained acute kidney injury (AKI); timely initiation of immune work-up and kidney biopsy becomes imperative. In this instance, the presence of positive ANCA, anti-MPO-ANCA, and ANA alongside DAH and idiopathic AKI increased the suspicion for AAV, prompting the consideration of renal biopsy as the most appropriate next step in diagnosis. While confirming DAH through bronchoscopy and/or PFTs would have been reasonable, the patient's care goals and limited functional reserve precluded the completion of these measures. Nonetheless, the patient underwent a renal biopsy, revealing pathology consistent with AAGN. The clinical presentation observed was most indicative of AAV linked to hydralazine.

Due to the uncommon occurrence of PRS secondary to hydralazine-induced AAV, there is a shortage of strong, evidence-backed treatment recommendations. While certain insights have been gleaned from case reports, the absence of dedicated guidelines for addressing hydralazine-induced AAV means that treatment decisions primarily hinge on established protocols for idiopathic ANCA vasculitis. In addition to the immediate discontinuation of hydralazine, this typically involves immunosuppressive measures using corticosteroids and immunomodulatory agents (methotrexate or mycophenolate mofetil in non-life-threatening AAV; cyclophosphamide or rituximab in life-threatening AAV) [7,15,16]. Plasmapheresis may be employed in severe cases (e.g., life- or organ-threatening, creatinine greater than 5.7 mg/dL, severe DAH) [7]. In this case, even with all available treatments, the patient succumbed to worsening hypoxia and persistent hemoptysis.

## Conclusions

Hydralazine-induced AAV leading to DAH is extremely rare, but the exact incidence or prevalence of this condition is unknown. As described in this case, general internists should have a high level of suspicion for hydralazine-induced AAV causing DAH in the setting of hydralazine use, AKI, new-onset anemia, hemoptysis, and hypoxemia. Given the high associated mortality in cases of PRS, prompt recognition of the condition, cessation of hydralazine, initiation of immunosuppression, and early plasma exchange are essential to an improved prognosis.
